# cDC1 Dependent Accumulation of Memory T Cells Is Required for Chronic Autoimmune Inflammation in Murine Testis

**DOI:** 10.3389/fimmu.2021.651860

**Published:** 2021-07-26

**Authors:** Yuchao Jing, Min Cao, Bei Zhang, Xuehui Long, Xiaoming Wang

**Affiliations:** Department of Immunology, Key Laboratory of Immune Microenvironment and Diseases, State Key Laboratory of Reproductive Medicine, Nanjing Medical University, Nanjing, China

**Keywords:** testis, T cells, dendritic cell, spermatogenesis, chronic autoimmune orchitis

## Abstract

As an immune privilege site, there are various types of immune cells in the testis. Previous research has been focused on the testicular macrophages, and much less is known about the T cells in the testis. Here, we found that T cells with memory phenotypes were the most abundant leukocyte in the testis except for macrophages. Our results showed that the proportion of testicular T cells increases gradually from birth to adulthood in mice and that the primary type of T cells changed from γδTCR^+^ T cells to αβTCR^+^ T cells. In addition, under homeostatic conditions, CD8^+^ T cells are the dominant subgroup and have different phenotypic characteristics from CD4^+^ T cells. We found that cDC1, but not cDC2, is necessary for the presence of T cells in the testis under physiological state. A significant decrease of T cells does not have a deleterious effect on the development of the testis or spermatogenesis. However, cDC1-dependent T cells play an indispensable role in chronic autoimmune orchitis of the testis. Collectively, our multifaceted data provide a comprehensive picture of the accumulation, localization, and function of testicular T cells.

## Introduction

Testis is the male reproductive gland, composed of seminiferous tubules and interstitial cells. Seminiferous tubules are the places where spermatogenesis occurs. Testicular interstitium is the connective tissue between the seminiferous tubules, and multiple types of cells reside in the intersitium, including Leydig cells, fibroblasts, and a large population of immune cells (1, 2). Testicular macrophages are the main leukocytes and perform multiple functions during development and adulthood (3–5). However, the other immune cells in the testis, especially T cells, have not been adequately studied.

T cell is an essential part of the adaptive immune system (6). Immune responses commence when naïve T cells encounter antigen and costimulatory ligands presented by DC, resulting in interleukin 2 (IL-2) production, proliferation, and differentiation to effector cells. Activated effector cells are short-lived, but a proportion of them survive as memory T cells, which will produce faster, larger, and more effective responses to antigens encountered again (7, 8). In addition, previous studies have shown that a large fraction of memory T cells in tissues comprise a distinct subset, designated tissue resident memory T (TRM) cells (9, 10). Functionally, TRM cells mediate rapid *in situ* protection against pathogen infections and are more effective than circulating memory T cells in pathogen clearance (11, 12). Moreover, studies have revealed that TRM also plays an important role in inflammatory autoimmune diseases, especially skin and CNS-associated diseases (13). TRM-targeting treatments have shown promising therapeutic effects on mouse model of vitiligo disease (14). Interestingly, mouse studies show that TRM cells may accumulate naturally with age in the CNS (15). However, as an immune privilege site, the development, maintenance, and physiological or pathological function of T cells in the testis have not been fully characterized.

Here, we examined the accumulation, localization, and function of T cells in the testis and analyzed their characteristic phenotype through flow cytometry analysis combined with scRNA-seq. Based on our results, T cells comprise a large proportion of immune cells in the testis, and most of these T cells adopt TRM phenotype. cDC1, but not cDC2, is necessary for the presence of T cells in the testis. We found that a significant decrease of T cells did not have a deleterious effect on the development of the testis or spermatogenesis. Finally, we pointed out that cDC1-dependent T cells play a critical role in chronic autoimmune inflammation of the testis.

## Materials and Methods

### Animals

*Rosa26-LSL-Tomato* (JAX 007909), *Lyz2-Cre* (JAX 004781), *CD4-Cre* (JAX017336), *BATF3* ^−/−^ (JAX 013755), *IRF4* ^fl/fl^ (JAX 009380), and *CD11c-Cre* (Itgax-Cre, JAX 008068) mice were obtained from the Jackson Laboratory. C57BL/6 mice were purchased from Animal Core Facility of Nanjing Medical University. One- to 16-week-old male mice were used for experiments. Mice were bred and housed under controlled environmental conditions (12 h light/12 h darkness, temperature range of 20–23°C, relative humidity to 50–60%) and under specific pathogen-free conditions. Animal experiments were approved by the Institutional Animal Care and Use Committee (IACUC) of Nanjing Medical University.

### Cell Isolation

Testis leukocytes were obtained as described previously with modifications (16). In brief, the testes were decapsulated and digested with 0.5 mg/ml type I collagenase (Sigma), 0.1 mg/ml DNase I (Sigma-Aldrich), and 2% FBS in RPMI^-^1640 at 180 rpm, 37°C for 20 min. Cell suspensions were passed through 100-μm cell strainer cap to remove the seminiferous tubules. After washing twice with ice-cold PBS, the cells were subjected to flow cytometry.

### Antibodies and Flow Cytometry

For analysis of surface markers, cells were stained in PBS containing 2% fetal bovine serum (FBS) with antibodies as indicated. Foxp3 staining was performed according to the manufacturer’s instructions (eBioscience). To determine cytokine expression, cells were stimulated with phorbol12-myristate 13-acetate (PMA), ionomycin, and monensin for 5 h. At the end of stimulation, cells were stained with the indicated antibodies according to the manufacturer’s instructions (eBioscience). Unspecific binding to low-affinity Fc receptors was blocked by incubating the cells with unconjugated CD16/32 antibody (BioXcell; clone 2.4G2). The following antibodies were used for flow cytometry: CD45-AlexaFluor 700 (BioLegend; clone 104), CD45-PE (BioLegend; clone 104), CD11b-APC/Cy7 (BioLegend; clone M1/70), F4/80-PerCP/Cy5.5, CD3ϵ-APC (BioLegend; clone 145-2C11), TCRβ-PerCP/Cy5.5 (eBioscience; clone H57-597), γδTCR-FITC (BD; clone GL3), CD4-APC/Cy7 (BD; clone GK1.5), CD8α-PerCP/Cy5.5 (BioLegend; clone 53-6.7), CD44-PE/Cy7 (BioLegend; clone IM7), CD62L-APC (eBioscience; clone 17-0621-82), CD103-biotin (BD; clone M209), CD69-FITC (BioLegend; clone H1.2F3), NK1.1-FITC (eBioscience; clone PK136), CD19-APC (BioLegend; clone 6D5), MHCII-APC (BioLegend; clone AF6-120.1), CD11c-PE/Cy7 (BD; clone HL3), CD24-PE (BioLegend; clone M1/69), and Streptavidin-v450 (BD; cat #560797). DAPI (eBioscience, cat #62247) or Fixable Viability Dye eFluor 780 (eBioscience, cat #65-0865-18) was used to distinguish the dead and live cells.

### Intravenous Staining of CD45^+^ Cells Localized in the Vasculature of the Testis

Intravenous staining of CD45^+^ cells was carried out as previously described. For intravascular cell staining of CD45^+^ cells, mice were intravenously injected with 2 μg anti-CD45-PE antibody (BioLegend; clone 104) diluted in 100 μl PBS, 3 min before sampling.

### Cryosection Immunofluorescence

For immunostaining using frozen sections, testes were embedded in optimum cutting temperature compound (O.C.T.) and frozen in liquid nitrogen. Serial sections were cut at 5 μM thickness onto glass slides and stored at −80°C until use. Slides were blocked 1 h at room temperature in PBS/5% BSA/1% mouse serum/1% anti-CD16/32 (BioXcell; clone 2.4G2). Primary and secondary antibodies were added, followed by staining with DAPI. The following antibodies were used throughout this study: anti-F4/80-AlexaFluor647 (BioLegend; clone BM8), CD3ϵ (Santa Cruz Biotechnology; clone M-20), SOX9 (abcam; clone EPR14335-78), Biotin-SP-conjugated Affinipure Donkey Anti-Goat IgG (H+L) (Jackson immunoresearch; code:705-065-147), Cy™3 conjugated Sreptavidin (Jackson immunoresearch; code:016-160-084), Cy™3 AffiniPure Donkey Anti-Rabbit IgG (H+L) (Jackson immunoresearch; code: 711-165-152).

### Induction of Experimental Autoimmune Orchitis (EAO)

WT and *BATF3^−/−^* male mice were used in EAO induction based on previously described method in a mice model (17). Testicular homogenate (TH) was prepared from decapsulated testes collected from adult syngeneic mice and homogenized in sterile PBS at a ratio of 1:1. Mice were immunized three times every 14 days with a mixture of TH in Complete Freund’s adjuvant (CFA; Sigma-Aldrich, lot #SLBR3877V) at a ratio 1:1, followed by an i.v. injection of 100 ng Bordetella pertussis toxin (Calbiochem) in 100 μl PBS. Each mouse was subcutaneously injected in three sites near the popliteal lymph nodes with a total volume of 200 μl. Adjuvant control animals received CFA mixed with PBS instead of testicular homogenate following the same scheme. Testes were collected for EAO analysis at 50 days after the first immunization.

### Computer Assisted Sperm Analysis (CASA)

Motility parameters of epididymis semen were performed by Computer Assisted Sperm Analysis System (CASA, Hamilton Thorne IVOS). The chambers heated at 37°C were filled with 10 μl of sample (approximately 1 × 10^7^ cells/ml), and five fields were selected for the analysis. The following parameters were evaluated: Sperm count (M/ml), motility cell (%), progressive cell (%), mean average velocity (VAP, μm/s), curvilinear velocity (VCL, μm/s), and straight line velocity (VSL, μm/s).

### Cell Sorting and Single-Cell RNA-Seq Analysis

For scRNA-seq analysis, testicular immune cells were isolated as above and were sorted CD45^+^ cells with BD FACS. Then, CD45^+^ scRNA-seq analysis was conducted using the BD Rhapsody Single-Cell Analysis System (BD Biosciences), following the manufacturer’s protocol. In short, the single-cell suspension was loaded into a BD Rhapsody cartridge with >200,000 microwells, and single-cell capture was achieved by random distribution and gravity precipitation. Beads with oligonucleotide barcodes were added to saturation so that a bead was paired with a cell in a microwell. The cells were lysed in the microwell cartridge to hybridize mRNA molecules to barcoded capture oligos on the beads. Beads were collected into a single tube for reverse transcription. Upon cDNA synthesis, each cDNA molecule was tagged on the 5′ end (that is, the 3′ end of a mRNA transcript) with a molecular index and cell label indicating its cell of origin. Whole transcriptome libraries were prepared using the BD Resolve single-cell whole-transcriptome amplification workflow. In brief, second-strand cDNA was synthesized, followed by ligation of the adaptor for universal amplification. Eighteen cycles of PCR were used to amplify the adaptor-ligated cDNA products. Sequencing libraries were prepared using random priming PCR of the whole-transcriptome amplification products to enrich the 3′ end of the transcripts linked with the cell label and molecular indices. The BD Rhapsody Analysis Pipeline was used to process the sequencing data (fastq files), and output result files were analyzed and visualized using the BD Data View software (BD Biosciences). The raw data have been uploaded to Gene Expression Omnibus public database (GSE152232).

The core TRM cell and TCM (central memory) cell signatures were generated by integrating differential expression (>1.5 fold change) data comparing TRM cells from the following tissues to TCM cells: day 35 IELs (LCMV) (18), day 35 kidney parenchyma (LCMV), day 30 skin CD103+CD8+ (herpes simplex virus), day 30 lung CD103+CD8+ (influenza virus) (19), and day 20 CD103+ brain (vesicular stomatitis virus) (20); overlapping genes upregulated in all TRM cell populations were defined as the core tissue-residency signature (107 genes), and genes upregulated in TCM populations were defined as the TCM signature (73 genes).

### Quantification and Statistical Analysis

Unless specified, data were presented as the mean ± standard deviation(SD). Samples were analyzed using unpaired t test for two groups and ANOVA for multiple groups. P values are denoted in figures by: NS, not significant; *, p < 0.05; **, p < 0.01; ***, p < 0.001.

## Result

### The Immune Cell Composition in Adult Mouse Testis

**Figure 1:**
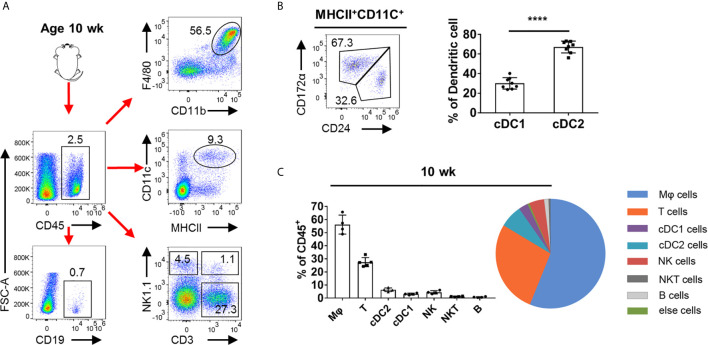
FACS analysis of the immune cells in adult mouse testis. **(A)** Representative FACS plots showing the gating strategies for CD45^+^ cell, Mφ (CD11b^+^F4/80^+^), DC (CD11c^+^MHCII^+^), T cells (CD3^+^NK1.1^−^), NK cells (NK1.1^+^CD3^−^), NKT cells (CD3^+^NK1.1^+^), and B cells (CD19^+^) from the testis (pre-gated on single cell/live cell). **(B)** FACS analysis of the frequency of cDC1 (CD24^+^CD172α^−^) and cDC2 (CD172α^+^CD24^−^) in total DC in the testis of C57BL/6 mice. n = 8. Data were pooled from two experiments. **(C)** Summary graphs and pie chart illustrate the percentage of Mφ cells, T cells, cDC1 cells, cDC2 cells, NK cells, NKT cells, and B cells in CD45^+^ cell from the testis of 10-week-old mice. Error bars are the mean ± SD. **(B)** One-way ANOVA with Tukey’s multiple comparisons test was performed. ****P < 0.0001.

To analyze the overall leukocyte landscape in the adult testis by flow cytometry, we first gated CD45^+^ cells and then analyzed the presence of immune cell populations (for the gating strategy, see **Supplementary Figure 1A**). As previous reported, TMφ (Testicular macrophage) are indeed the major immune cell subsets (21, 22). Moreover, there are many other leukocytes in the testicular interstitium, including DCs, NK cells, T cells, NKT cells, and few B cells (**Figure 1A**). For testicular dendritic cells, we found that the CD172α^+^CD24^−^ dendritic cells (cDC2) outnumbered the CD24^+^ CD172α^−^ (cDC1) cells (**Figure 1B**). NK and NKT cells account for a small part in testicular leukocytes (**Figure 1C**). Of note, we found that T cells were the most abundant leukocyte in the testis except for macrophages, which aroused our great interest (**Figure 1C**).

### Dynamic Accumulation and Localization of T Cells in the Testis

**Figure 2:**
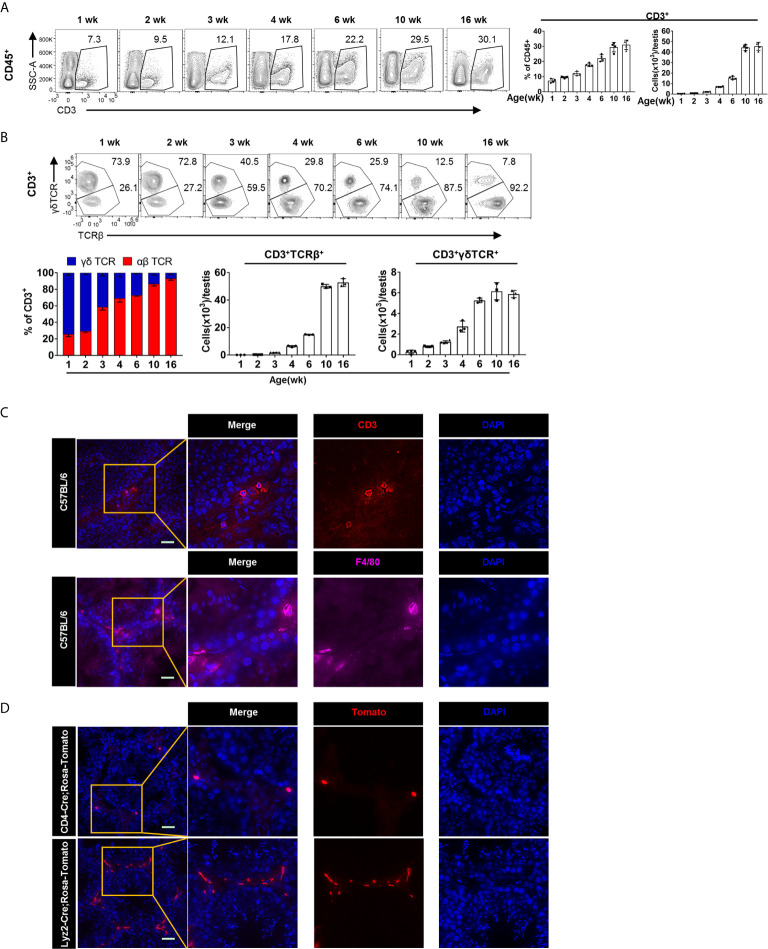
The dynamic changes and localization of T cells in postnatal testis. **(A)** FACS analysis of the frequency and number of T cells (CD3^+^) in the testis from the 1-, 2-, 3-, 4-, 6-, 10-, and 16-week-old C57BL/6 mice. **(B)** FACS analysis of the frequency and number of γδTCR^+^ (CD3^+^γδTCR^+^) and αβTCR^+^ (CD3^+^TCRβ^+^) T cells in the testis from the 1-, 2-, 3-, 4-, 6-, 10-, and 16-week-old C57BL/6 mice. **(A, B)** n = 4–5. Data are representative of two experiments. Error bars are the mean ± SD. **(C)** Microscopic analyses of CD3 and F4/80 in frozen sections of the testis from 10-week-old C57BL/6 mice. **(D)** Frozen sections of the testis from *CD4-Cre*; *Rosa-Tomato* mice and *Lyzm2-Cre*; *Rosa-Tomato* mice. Expression of Tomato protein represents T cells and macrophages, respectively. **(C, D)** Scale bars: 50 μm.

To track the dynamic changes of T cell in postnatal testis, we used 1-, 2-, 3-, 4-, 6-, 10-, and 16-week-old mice for flow cytometry analysis (for the gating strategy, see **Supplementary Figure 1A**). We found that the proportion and number of CD3^+^ T cells in testis increased with age (**Figure 2A**). Furthermore, we found that in testis from mice younger than 3 weeks, the γδTCR^+^ T cell outnumbered the αβTCR^+^ T cells, whereas the αβTCR^+^ T cells had become the predominant population in mice older than 3 weeks (**Figure 2B**). Interestingly, such age-associated gradual decrease of γδTCR ^+^ T cells and the increase of αβTCR^+^ T cells tended to be stable after adulthood (**Figure 2B**). Among αβTCR^+^ T cells population, there were three subpopulations: CD4^−^CD8^−^, CD4^+^CD8^−^, and CD4^−^CD8^+^ (**Supplementary Figure 2A**). The proportion of the three αβ T cell populations showed dynamic changes. As mice aged, the dominant T cell subpopulation changed from CD4^−^CD8^−^ to CD4^+^CD8^−^ and CD4^−^CD8^+^ cells (**Supplementary Figure 2B**). In addition, contrary to lymphoid organs, there were more CD8^+^ T cells than CD4^+^ T cells in the testis, and the proportion of both cells increased with age (**Supplementary Figure 2B**). The abundant presence of CD4^−^CD8^−^ αβ T cell subset seemed also unique to the testis, whereas very few such cells were observed in the spleen (**Supplementary Figure 3A**). On the other hand, we found that there were a large number of γδTCR ^+^CD4^−^CD8^+^ T cells in the testis before 4 weeks old, whereas the γδTCR^+^ T cells were almost exclusively CD4^−^CD8^−^ after 4 weeks old (**Supplementary Figures 2A, C**).

In order to directly observe the localization of T cells in the testis, cryosection of the testis from 10-week-old C57BL/6 mice were stained for T cells with anti-CD3 antibody. We found that T cells are evenly distributed in the testicular interstitium (**Figure 2C**). As a positive control, we also stained for TMφ, with F4/80 (**Figure 2C**). As previously reported, there are two types of TMφ according to their morphology. The peritubular macrophages with elongated morphology embrace the gamete-producing seminiferous tubules, whereas the interstitial macrophages reside in the testicular interstitium between the tubules (16, 23). Meanwhile, we can see that there are more TMφ than T cell in the testis. One caveat for antibody staining is the potential unspecificity. Therefore, to further verify the abundant presence of T cells, we used cell-fate mapping mice. *CD4-Cre* and *Lyz2-cre* mice were crossed onto *Rosa26-LSL-Tomato* mice, respectively. The T cells and TMφ in the testis of offspring mice were thus labeled as tdTomato^+^. Consistent results as above immunofluorescence were observed (**Figure 2D** and **Supplementary Figure 2D**).

### The Memory Phenotype of T Cells in the Testis

**Figure 3:**
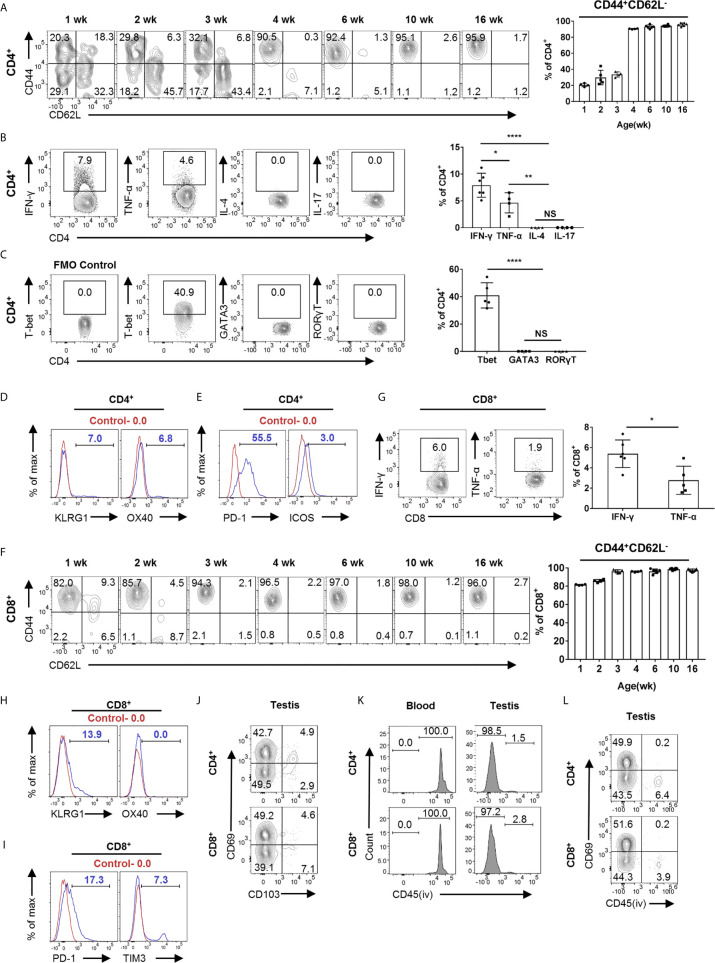
Heterogeneity of T cells in the testis revealed by FACS analysis. **(A)** FACS analysis of the frequency of memory (CD44^+^CD62L^−^) CD4^+^ T cells in the testis from the 1-, 2-, 3-, 4-, 6-, 10-, and 16-week-old C57BL/6 mice. **(B)** FACS analysis of the percentage of IFN-γ-, TNF-α-, IL-4-, and IL-17-producing CD4^+^ T cells in the testis of C57BL/6 mice. **(C)** FACS analysis of the percentage of T-bet^+^, GATA3^+^, and RORγT^+^ CD4^+^ T cells in the testis of C57BL/6 mice. **(D, E)** Representative FACS data of PD-1, ICOS, OX40, and KLRG1 expression on CD4^+^ T cells in the testis of C57BL/6 mice. **(F)** FACS analysis of the frequency of memory (CD44^+^CD62L^−^) CD8^+^ T cells in the testis from the 1-, 2-, 3-, 4-, 6-, 10-, and 16-week-old C57BL/6 mice. **(G)** FACS analysis of the percentage of IFN-γ- and TNF-α-producing CD8^+^ T cells in the testis of C57BL/6 mice. **(H, I)** Representative FACS data of PD-1, TIM3, OX40, and KLRG1 expression on CD8^+^ T cells in the testis of C57BL/6 mice. **(J)** A representative FACS plot of CD69 and CD103 expression on CD4^+^ and CD8^+^ T cells in the testis of C57BL/6 mice. **(K)** Mice were intravenously injected with 2 μg anti-CD45 PE antibody diluted in 100 μl PBS. Blood and testis were collected 3 min later for analysis. FACS analysis of the percentage of CD45PE^+^ cells in CD4^+^CD69^+^ and CD8^+^CD69^+^ cells from blood and testis. **(L)** Mice were intravenously injected with 2 μg anti-CD45 PE antibody diluted in 100 μl PBS, and testis were collected 3 min later for analysis. FACS analysis of the percentage of CD45PE^+^ cells of CD4^+^ and CD8^+^cells from the testis. **(A–C, F, G)** n = 4–6. Data are representative of two experiments. Error bars are the mean ± SD. One-way ANOVA with Tukey’s multiple comparisons test for **(B, C)** and unpaired t test for **(G)**. *P < 0.05; **P < 0.01; ****P < 0.0001; NS, not significant.

We next phenotypically characterized T cells in the testis. First, we analyzed the subsets of CD4^+^ T cells, including CD4^+^ naïve, memory, T-helper 1 (TH1), 2 (TH2), 17 (TH17), Regulatory T cells (Treg), and CD4^+^ exhausted T cells (Tex). Notably, we found that more than 90% of T cells are memory T cells (CD44^+^CD62L^−^) in the testis of adult mice, which is distinct from lymphoid organs where memory T cells account for only a small proportion (**Supplementary Figure 3B**). Interestingly, CD4^+^ T cells acquired such memory phenotype gradually with age (**Figure 3A**). Furthermore, we analyzed the expression of cytokines and found that CD4^+^ T cells secreted IFN-γ and TNF-α, but not IL-4 and IL-17 (**Figure 3B**). Therefore, we speculate that there are TH1 cells instead of TH2 and TH17 cells in the testis. A similar result was obtained by analyzing the expression of transcription factors (**Figure 3C**). Interestingly, although most of the CD4^+^ T cells in the testis are memory cells, only a small proportion of them expressed IFN-γ, far below than their splenic counterparts (**Supplementary Figure 3D**). Further, we found that CD4^+^ T cells express OX40 and KLRG1 at low levels, and these makers are mainly expressed on the surface of effector T cell subsets (**Figure 3D**). Next, we analyzed Tex markers. Interestingly, we found that CD4^+^ T cells express high levels of PD1, low levels of ICOS (**Figure 3E**), but no detectable level of Tim3, LAG3, and CTLA4 (data not shown). The high level of PD1 expression seemed to be unique to CD4^+^ memory T cells in the testis, since splenic memory CD4^+^ T cells express much lower level of PD1 (**Supplementary Figure 3E**). Additionally, Tregs, as immunosuppressive cells, account for about 15% of CD4^+^ T cells in the testis, similar as its frequency in spleen (**Supplementary Figure 3C**).

Next, we analyzed CD8^+^ T cell. Different from CD4^+^ T cells in the testis, more than 80% of CD8^+^ T cells in 1-week-old mice are already memory T cells, and the proportion of memory CD8^+^ T cells gradually increased to more than 90% with age (**Figure 3F**). In addition, compared to splenic counterparts, we found that memory CD8^+^ T cells in the testis also secrete lower levels of cytokines (**Figure 3G** and **Supplementary Figure 3D**). For effector T cell maker, CD8^+^ T cells express a certain level of KLRG1 but not OX40 (**Figure 3H**). To our surprise, unlike CD4^+^ T cells, CD8^+^ T cells in testes expressed lower levels of PD1 and a low level of TIM3 (**Figure 3I**), but no detectable level of LAG3, ICOS, and CTLA4 (data not shown).

TRM is a recently defined subset of memory T cells, which resides in non-lymphoid tissues to mediate immediate immune protection and is also involved in inflammatory diseases (9, 11, 24, 25). CD69, which antagonizes S1pr1 egressing signal, is the predominant marker for TRM cells, and CD103 is also expressed on some of TRM cells, depending on distinct tissue contexts (9). FACS analysis showed that 40–50% of T cells were CD69^+^, with a small proportion as CD103^+^, suggesting that these cells are TRM cells (**Figure 3J**). To analyze whether these cells are tissue resident, we performed *in vivo* labeling experiments. The mice were intravascularly injected with CD45-PE antibody and sacrificed 3 min later to distinguish between tissue-localized cells and bloodborne cells (26, 27). We found that the majority of CD69^hi^ T cells (both CD4^+^ and CD8^+^) in the testis were PE negative, whereas as a control, the T cells in blood were all PE positive, suggesting that these CD69^+^ T cells in the testis were indeed TRM cells (**Figures 3K, L**). Together, our data reveal that most of the T cells in the testis obtain memory phenotype, and about half of these cells are TRM cells.

### Single-Cell RNA-Seq Reveals the Heterogeneity of T Cells

To gain comprehensive insight into which types of immune cells are present in the testicular interstitium and reveal the heterogeneity of T cells, we performed single-cell transcriptomics analysis (scRNAseq). As the most abundant immune cells, TMφ have been intensively and thoroughly studied previously. We performed a percoll-based gradient centrifugation to remove most of the TMφ, so that we can focus more on other cells, including T cells. Cell partitioning *via* t-distributed stochastic neighbor embedding (tSNE) analyses identified 10 clusters. Cluster identity was assigned based on known cell-type marker expression. Multiple subsets of immune cells were identified in the mouse testicular interstitium, including macrophages, T cells, NKT cells, dendritic cells, granulocytes, few B cells, etc. (**Supplementary Figure 4A**).

**Figure 4:**
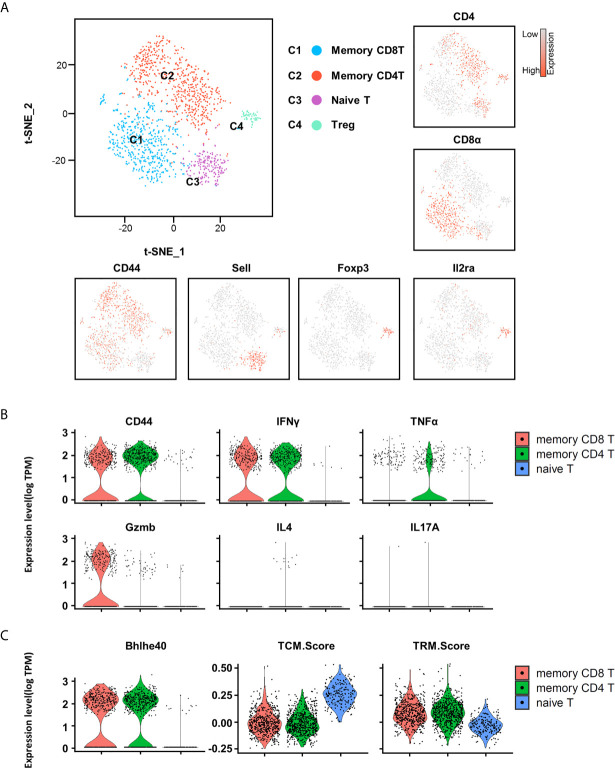
Heterogeneity of T cells in testis revealed by scRNA-seq analysis. **(A)** t-distributed stochastic neighbor embedding (tSNE) and clustering analysis of combined single-cell transcriptome data of testicular immune cells from C57BL/6 mice (a single experiment with a sample containing cells pooled from 10 mice). Each dot represents a single cell and is colored according to its cluster identity as indicated on the figure key. The four-cluster identities were assigned based on marker gene expression. **(B)** The violin plots show the expression levels of CD44, IFN-γ, TNF-α, Gzmb, IL4, and IL17A on memory CD8^+^ T cells, memory CD4^+^ T cells, and naïve T cells. **(C)** The violin plots show the expression levels of TCM (central memory) and TRM cell signatures on memory CD8^+^ T cells, memory CD4^+^ T cells, and naïve T cells.

To further understand the heterogeneity of the T cell population, we used unsupervised cluster analysis, and the T cells can be further partitioned into four clusters on the basis of their character maker (**Figure 4A**). Consistent with the FACS results, CD44 transcripts were detected in memory T cells except the minor naive T cell cluster. IFN-γ and TNF-α but not IL-4 and IL-17A transcripts were present in memory T cells. Compared with memory CD8^+^ T, TNF-α transcription is more abundant in memory CD4^+^ T cells, which is consistent with the FACS results (**Figure 4B**). Interestingly, Granzyme B (GZMB) transcripts were present in memory CD8^+^ T cells, indicating that these cells retain effector function (**Figure 4B**). Of note, transcription factor Bhlhe40 has previously been identified as an important transcription factor that promotes TRM development and polyfunctionality by sustaining TRM cell mitochondrial fitness and functional epigenetic state (28). Here, we also found that the transcription factor Bhlhe40 was highly expressed in memory T cells, suggesting that these T cells are indeed TRM cells (**Figure 4C**). To further confirm the abundant presence of TRM cells in the testis, we constructed a core TRM cell and TCM cell transcriptional signature by computational integration of public TRM gene-expression datasets from small intestine IELs (18) (intraepithelial lymphocytes), kidney, lung (19), skin (19), and brain (20), to evaluate the T cells in the testis (signature genes are listed in **Supplementary Table 1**). Notably, we found that the core TRM signature gene set was highly expressed in memory CD4^+^ and memory CD8^+^ T cells (**Figure 4C**). Conversely, the core TCM signature was enriched in naive T cells (**Figure 4C**). Therefore, by scRNAseq, we confirmed that most of the T cells in the testis were memory T cells, and TRM cells were abundantly present.

### cDC1, but Not cDC2, Is Necessary for the Presence of T Cells in the Testis

DCs have long been recognized as the most potent antigen-presenting cells to activate T cells. Conventional DCs (cDCs) are composed of distinct subsets, with each subset specifically regulated by distinct transcriptional factors. For example, interferon regulatory factor 4 (IRF4) and Notch2 are critical for cDC2s generation, whereas IRF8 and BATF3 are essential for cDC1s development (29).

**Figure 5:**
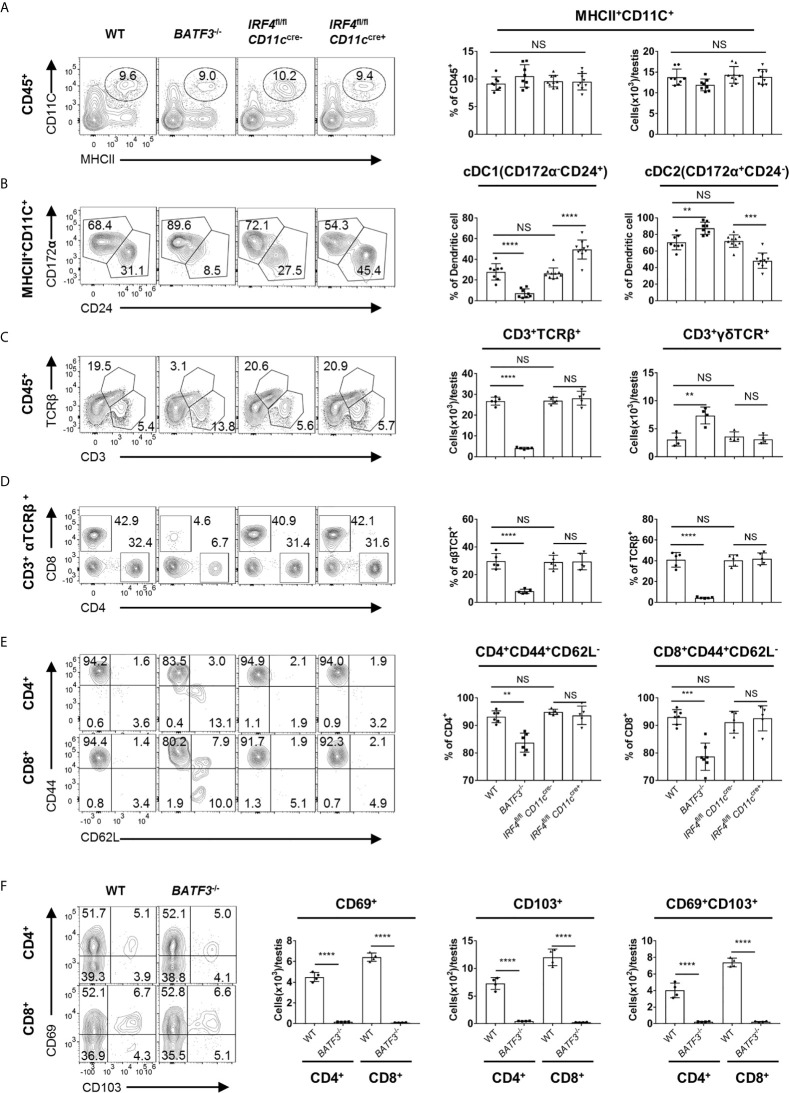
cDC1, but not cDC2, is necessary for the presence of T cells in the testis. **(A)** FACS analysis of the number and frequency of DC (MHCII^+^CD11C^+^) in the testis from 8-week-old WT, *BATF3* ^−/−^, *IRF4* ^fl/fl^ *CD11c* ^cre−^, and *IRF4* ^fl/fl^ *CD11c* ^cre+^ mice. **(B)** FACS analysis of the frequency of cDC1 (CD24^+^CD172α^−^) and cDC2 (CD172α^+^CD24^−^) cells in total DC of the testis from 8-week-old WT, *BATF3* ^−/−^, *IRF4* ^fl/fl^ *CD11c* ^cre−^, and *IRF4* ^fl/fl^ *CD11c* ^cre+^ mice. **(A, B)** n = 8–10. Data were pooled from two experiments (WT and *BATF3* ^−/−^ mice) and three experiments (*IRF4* ^fl/fl^ *CD11c* ^cre−^ and *IRF4* ^fl/fl^ *CD11c* ^cre+^ mice). **(C)** FACS analysis of the frequency and number of αβTCR^+^ T (CD3^+^TCRβ^+^) in the testis from 8-week-old WT, *BATF3* ^−/−^, *IRF4* ^fl/fl^ *CD11c* ^cre−,^ and *IRF4* ^fl/fl^ *CD11c* ^cre+^ mice. **(D)** FACS analysis of the frequency of CD4^+^CD8^−^ and CD4^−^CD8^+^ T cells in CD3^+^TCRβ^+^ of the testis from 8-week-old WT, *BATF3* ^−/−^, *IRF4* ^fl/fl^ *CD11c* ^cre−^, and *IRF4* ^fl/fl^ *CD11c* ^cre+^ mice. **(E)** FACS analysis of the frequency of memory (CD44^+^CD62L^−^) CD4^+^ and CD8^+^ T cells in the testis from 8-week-old WT, *BATF3* ^−/−^, *IRF4* ^fl/fl^ *CD11c* ^cre−^, and *IRF4* ^fl/fl^ *CD11c* ^cre+^ mice. **(C–E)** n = 5–6. Data are representative of two experiments. Error bars are the mean ± SD. One-way ANOVA with Tukey’s multiple comparisons test for **(A–E)** and unpaired t test for **(F)**. **P < 0.01; ***P < 0.001; ****P < 0.0001; NS, not significant.

To evaluate the contribution of dendritic cells to the accumulation and maintenance of T cells in the testis, we analyzed *BATF3* ^−/−^ and *IRF4* ^fl/fl^ *CD11c* ^cre+^ mice, which have been widely used as cDC1- and cDC2-deficient mouse models, respectively (30–38). Although there was no significant change in the proportion or quantity of total DC in testis (**Figure 5A**), we observed significantly reduced proportion of cDC1 and cDC2 in the testis and spleen of *BATF3* ^−/−^ mice and *IRF4* ^fl/fl^ *CD11c* ^cre+^ mice, respectively (**Figure 5B** and **Supplementary Figure 5A**). The reduction of cDC1 or cDC2 had no effect on T cell frequency and number in lymphoid organs (**Supplementary Figure 5B**). However, when examining the T cells in the testes, we found that the proportion and number of αβTCR^+^T cells in the testes of *BATF3* ^−/−^ mice were strikingly reduced compared with that of control mice, whereas T cells in the testis from *IRF4* ^fl/fl^ *CD11c* ^cre+^ mice were unaffected at all (**Figure 5C**). γδT cells in the testes of *BATF3* ^−/−^ mice were slightly increased, which might be a compensatory effect, or secondary to the space saved by dramatically reduced αβT cells. Focused on αβ T cell subsets, we found that the proportion of CD4^+^CD8^−^, CD4^−^CD8^+^ T cell, and memory-like T-cell population were lower in the testes but not in the spleen of *BATF3* ^−/−^ mice than control mice (**Figures 5D, E**, and **Supplementary Figures 5B, C**). Furthermore, although the TRM percentage was not altered, the TRM cell number in testes of *BATF3^−/−^* was also strikingly reduced compared with control mice (**Figure 5F**). Importantly, the number of other immune cells, including macrophages, B cells, NK cells, and NKT cells, was not affected in the testes of *BATF3* ^−/−^ mice (**Supplementary Figures 6A–C**). Collectively, these data indicate that cDC1 rather than cDC2 is critical for the maintenance and activation of T cells in the testis.

### Reduced T Cells in the Testis Does Not Hinder the Testis Development and Spermatogenesis

**Figure 6:**
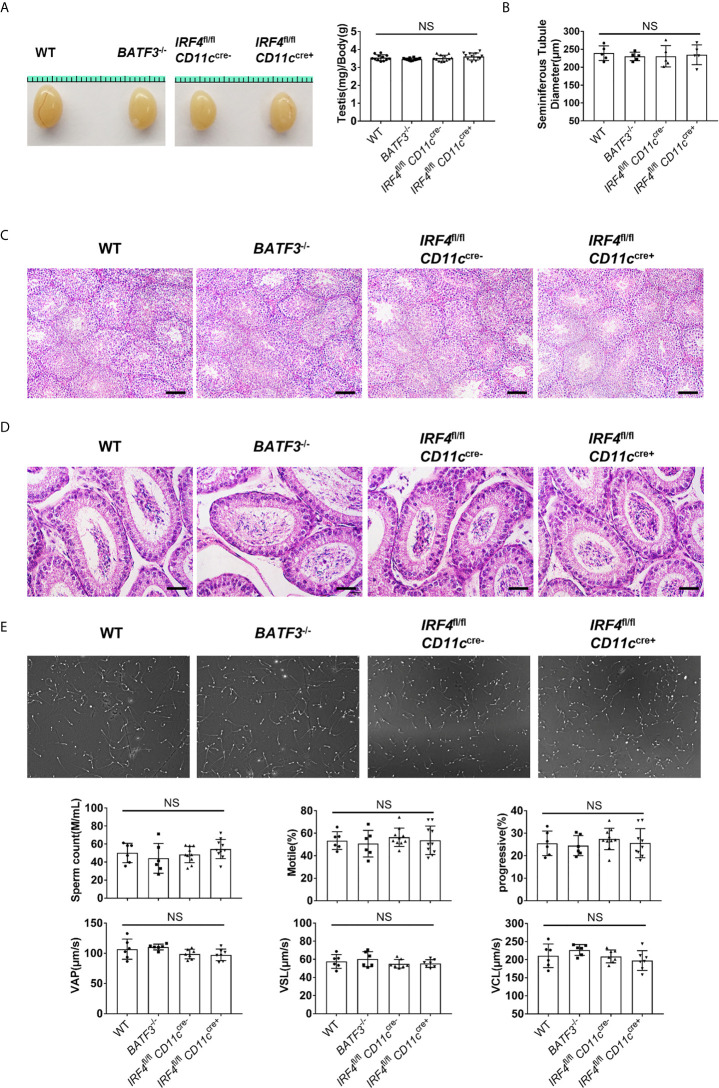
Reduced T cells in the testis does not hinder the testis development and spermatogenesis. **(A)** Gross morphology of representative testis from 8-week-old WT, *BATF3* ^−/−^, *IRF4* ^fl/fl^ *CD11c* ^cre−^, and *IRF4* ^fl/fl^ *CD11c* ^cre+^ mice, and relative weight of testis (testis weight (mg)/body weight (g)). n = 12. Data were pooled from two experiments. **(B)** Diameters of the seminiferous tubules of 8-week-old WT, *BATF3* ^−/−^, *IRF4* ^fl/fl^ *CD11c* ^cre−^, and *IRF4* ^fl/fl^ *CD11c* ^cre+^ mice. Each dot represents one mouse. n = 5. Data are representative of two experiments. **(C)** Hematoxylin-Eosin-stained testis sections from 8-week-old WT, *BATF3* ^−/−^, *IRF4* ^fl/fl^ *CD11c* ^cre−^, and *IRF4* ^fl/fl^ *CD11c* ^cre+^ mice. Scale bar: 100 μm. **(D)** Hematoxylin-Eosin-stained epididymis sections from 8-week-old WT, *BATF3* ^−/−^, *IRF4* ^fl/fl^ *CD11c* ^cre−^, and *IRF4* ^fl/fl^ *CD11c* ^cre+^ mice. Scale bar: 50 μm. **(E)** Sperm count, the ratio of motile sperm and progressive sperm, and the velocity of motile sperm [VCL (curvilinear velocity), VSL (straight line velocity), and VAP (average path velocity)] of indicated mice were detected by computer-assisted sperm analysis system. [n = 6 (WT, *BATF3* ^−/−^), 10 (*IRF4* ^fl/fl^ *CD11c* ^cre−^, *IRF4* ^fl/fl^ *CD11c* ^cre+^)]. Data are representative of two experiments. **(A, B, E)** Error bars are the mean ± SD. One-way ANOVA with Tukey’s multiple comparisons test was performed. NS, not significant.

Since the T cells in the testis increase dramatically as the testis matures, we then studied the functional importance of T cell for normal spermatogenesis. *BATF3* ^−/−^ mice showed greatly reduced T cells specifically in the testis; thus, it could serve as a mouse model to study the function of T cells in the testis. We found that the relative testis weights in *BATF3* ^−/−^ mice and *IRF4* ^fl/fl^ *CD11c* ^cre+^ were similar to that of the control mice (**Figure 6A**). We performed histological stainings and quantitative analyses of the testis, and the result showed that there were no detectable changes of tubular morphology and diameter (**Figure 6B** and **Supplementary Figure 7**). Despite the fact that the *BATF3* ^−/−^ mice lacked more than half of αβ T cells (**Figure 5C**) in the testis, they hosted normal spermatogenesis, containing a similar cellular density with full cohorts of differentiating germ cells in comparison to the control mice (**Figure 6C**). In line with these results, histological staining of caput epididymides showed normal presence of sperm in *BATF3* ^−/−^ mice (**Figure 6D**). Moreover, computer-assisted sperm analysis (CASA) can provide a simple and rapid quantitative assessment of the quality of mice sperm (39). Our CASA results showed that there was no difference in sperm count, the ratio of motile sperm and progressive sperm, and the velocity of motile sperm (VCL (curvilinear velocity), VSL (straight line velocity), and VAP (average path velocity)) among *BATF3* ^−/−^ mice, *IRF4* ^fl/fl^ *CD11c* ^cre+^ mice, and control mice (**Figure 6E**). Collectively, these analyses show that substantial reduction of T cell has no adverse effect on the development of the testis and normal spermatogenesis in mice.

### cDC1-Dependent T-Cell Accumulation Is Required for Chronic Autoimmune Inflammation in the Testis

Finally, we tested the role of testis T cells in chronic autoimmune orchitis model with *BATF3* ^−/−^ mice. Experimental autoimmune orchitis (EAO) is a useful model to study chronic autoimmune orchitis and infertility (40), and it is characterized by severe damage of seminiferous tubules with germ cells that undergo apoptosis and sloughing (41–43).

**Figure 7:**
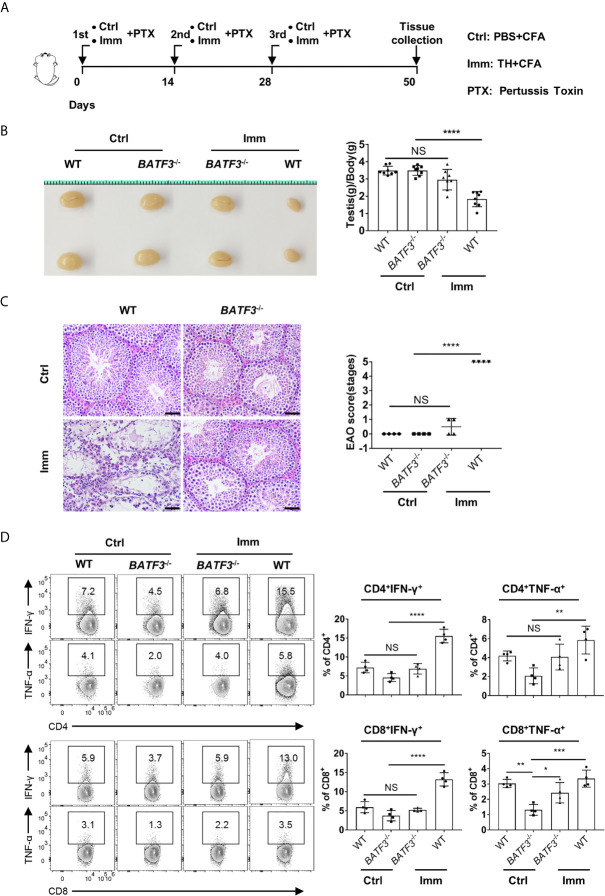
cDC1 and T cells play a more important role in experimental autoimmune orchitis. **(A)** The schema depicts the experimental procedures of experimental autoimmune orchitis. WT and *BATF3* ^−/−^ Mice were immunized three times with testicular homogenate (Imm group) or PBS (Ctrl group) emulsified in CFA. At 50 d after the first immunization, the testis was collected for further experiments. **(B)** Gross morphology of representative testis from control and immunization mice, and relative weight of testis [testis weight (mg)/body weight (g)]. n = 8. Data were pooled from two experiments. Error bars are the mean ± SD. **(C)** Hematoxylin-Eosin-stained testis sections from control and immunization mice. Scale bar: 50 μm. EAO scores were evaluated based on methods in the previous research. EAO was scored by five stages: stage 0, no detectable inflammation in the testis; stage 1, focal inflammation in the tunica albuginea; stage 2, focal inflammation adjacent to the tubuli recti; stage 3, inflammation surrounding the tubuli recti; stage 4, inflammation spreading around the seminiferous tubules and mild damage of the seminiferous epithelium; and stage 5, severe inflammation surrounding the tubules and damage of the seminiferous epithelium. **(D)** FACS analysis of the percentage of IFN-γ- and TNF-α-producing CD4^+^ and CD8^+^ T cells in the testis of indicated mice. **(C, D)** n = 4. Data are representative of two experiments. Error bars are the mean ± SD. One-way ANOVA with Tukey’s multiple comparisons test was performed. *P < 0.05; **P < 0.01; ***P < 0.001; ****P < 0.0001; NS, not significant.

In our mouse model of EAO, we found that the testis weight of immunized *BATF3* ^−/−^ mice was equivalent to that of the control, while the testis weight of the immunized WT mice was significantly reduced (**Figures 7A, B**), indicating that the *BATF3* ^−/−^ mice were resistant to EAO. Indeed, histological staining showed that the testis of immunized WT mice showed severe inflammation, manifested as atrophy of seminiferous tubules, shedding of seminiferous epithelium, and infiltration of a large number of lymphocytes in the stroma (**Figure 7C**). However, there was no obvious inflammation in the testes of immunized *BATF3* ^−/−^ mice (**Figure 7C**). In addition, the production of pro-inflammatory cytokine mediators did not increase in immunized *BATF3* ^−/−^ mice (**Figure 7D**). Blood–testis barrier, also known as the Sertoli cell seminiferous epithelium barrier, is a sign of immunological integrity within the testis (44). Compared with the control group, we observed that the Sertoli cells of WT mice in the immunized group were seriously disordered, while the Sertoli cells of *BATF3* ^−/−^ mice were only slightly disordered (**Supplementary Figure 8**). Collectively, the above results indicate that cDC1 and testis-accumulated T cells play a crucial role in chronic autoimmune orchitis.

## Discussion

The testis, the male gonad, has always attracted extensive research. However, as an immune privilege site, the research on testicular immune microenvironment mainly focuses on testicular macrophages (4, 45, 46), while other types of immune cells have not been studied in detail. Here, we found that T cells were the most abundant leukocyte in the testis except macrophages, which aroused our great interest.

Furthermore, we studied the accumulation, localization, and function of T cells in the testis. Our sc-RNAseq results also verified the heterogeneity of T cells. More interestingly, we found that cDC1 is necessary for the existence of T cells, while cDC2 is meaningless for the maintenance of T cells in the testis. The results under physiological condition showed that the decrease of T cells in the testis did not affect spermatogenesis. But to our surprise, the reduction of T cells greatly relieved the severity of chronic autoimmune orchitis. Overall, the above results explain the developmental map and function of T cells in the murine testis.

Here, we studied the development of T cells in the testis, and the results showed that the proportion of T cells in the testis increased with age. Interestingly, we found that more than 70% of the T cells that initially appeared in the testis were γδTCR^+^ T cells, and the proportion of αβTCR^+^ T cells gradually increased with age and eventually reached more than 90%. γδTCR^+^ T cells, which are innate-like T lymphocytes, are widely distributed in the peripheral blood and mucosal tissues (47). γδTCR^+^ T cells are mainly involved in the innate immune response, while αβTCR^+^T cells are mainly involved in the adaptive immune response (48). Depending on environmental signals, T cells are committed to effector or regulatory lineages with opposing functions (49). We speculate that the appearance of αβTCR^+^T cells may be related to spermatogenesis. With the development and maturation of the testis and the production of sperm cells, the appearance and proliferation of αβTCR+ T cells might effectively monitor the testicular ecological environment to ensure effective sperm production. Although we did not observe obvious spermatogenesis defect with *BATF3* ^−/−^ mice, further study under other contexts is still needed to draw a conclusion. In addition, during the development of αβTCR^+^ T cells, the proportion of CD4^−^CD8^−^ T cells decreased gradually, while the proportion of CD4^+^CD8^−^ and CD4^−^CD8^+^T cells increased gradually. This suggests that CD4^−^CD8^−^ T cells may be precursor cells, which give rise to both CD4^+^CD8^−^ and CD4^−^CD8^+^ T cells under physiological conditions with age.

In conclusion, our results provide for a picture of the accumulation and function of T cells in the testis. Our results indicate that the main type of T cells in the testis of mice will change from γδ TCR^+^ T cells to αβTCR^+^ T cells from birth to adulthood. In addition, T cells in the testes have unique phenotypic characteristics. Interestingly, under homeostatic conditions, CD8^+^ T cells are the dominant subsets and have different phenotypic characteristics from CD4^+^ T cells. Moreover, our scRNAseq analysis also verified the above results and revealed that the appearance and maintenance of T cells in testis were more closely related to DC. We then analyzed the T cells in the testis of *BATF3* ^−/−^ and *IRF4* ^fl/fl^ *CD11c* ^cre+^ mice and concluded that cDC1, not cDC2, is essential for the appearance and maintenance of T cells in the testis. However, the decrease of T cells in testis does not have harmful effects on testicular development and spermatogenesis under physiological conditions, while T cells play a more important role than macrophages and dendritic cells in chronic autoimmune orchitis.

## Data Availability Statement

The datasets presented in this study can be found in online repositories. The names of the repository/repositories and accession number(s) can be found below: NCBI [accession: GSE152232].

## Ethics Statement

The animal study was reviewed and approved by the Institutional Animal Care and Use Committee (IACUC) of Nanjing Medical University.

## Author Contributions

XW and YJ designed the experiment. YJ and BZ performed the experiment. MC and XL are involved in data processing. All authors contributed to the article and approved the submitted version.

## Funding

This work was supported by the National Key R&D Program of China (2018YFC1003900) and the Jiangsu Outstanding Young Investigator Program (BK20200030).

## Conflict of Interest

The authors declare that the research was conducted in the absence of any commercial or financial relationships that could be construed as a potential conflict of interest.

## Publisher’s Note

All claims expressed in this article are solely those of the authors and do not necessarily represent those of their affiliated organizations, or those of the publisher, the editors and the reviewers. Any product that may be evaluated in this article, or claim that may be made by its manufacturer, is not guaranteed or endorsed by the publisher.
