# Supplementary material for: cDC1 Dependent Accumulation of Memory T Cells Is Required for Chronic Autoimmune Inflammation in Murine Testis
Source: Front Immunol. 2021 Jul 26;12:651860. doi: 10.3389/fimmu.2021.651860 (PMC8350123; doi:10.3389/fimmu.2021.651860)
Supplement: Supplementary file 1 [file DataSheet_1.pdf]

Supplementary Table 1 Gene list for TRM and TCM signatures

	TRM signature	TCM signature
Gene name	Nr4a2 Itga1 Gpr34 Ell2 Serpinb9 Phlda1 Nr4a1 Fosl2 Coq10b Spats2 Ehd1 Slc3a2 Nr4a3 Cdh1 Fasl Bcl2a1c Dennd4a Osbpl3 Gzma Pmepa1 Smim3 Neurl3 Phactr2 Plscr1 Chn2 Egr1 Tigit Isy1 Tjp1 Gpr171 Rgs1 Hspa1a Egr2 Gch1 Baiap2 Odc1 Litaf Bhlhe40 Isg20 Il2ra Samsn1 Lrrc8d Sik1 Slc16a10 Spty2d1 Hilpda Hspa5 Hsp90aa1 Ctla4 Crem Tgif1 Tiparp Gla Il4ra Vps37b Adgrg1 Pdcd1 Tinf2 Icos Nfkbie Hpgds Qpct B4galnt4 Tnfrsf1b Arhgef12 Zfand5 Xcl1 Csrnp1 Skil Cwc25 Ern1 Hist1h1c Rgs2 Junb Abcb1b Btg2 D16Ertd472e Ptprj Gadd45b Gzmb Cish Il21r Ppp1r16b Pelo Irf4 Fgl2 Rnf149 Tob2 Hspa2 Slc16a6 Plk3 Per1 Pygl Jun Prdx6 Eif4e3	Eomes Samd3 Atp1b3 Rap1gap2 Fgf13 Ly9 Glipr2 Sell Tlr1 Elovl7 Xrn2 B3gnt5 Cd22 Klhdc1 S1pr1 Dnah8 Dapl1 Cd2ap Wfikkn2 Chil5 Pum3 Ms4a4c Usp33 Lpin1 Prkcq Rcbtb1 Spice1 Mboat1 Rasgrp2 Macf1 Arhgap15 Zfp459 Smpdl3b Sh3bp5 Wwp1 Sidt1 Tnfrsf26 Arhgap4 Racgap1 Lair1 Zfp825 Ttc13 Slamf6 Stk38 Pxylp1 Sh2d1a Arhgef18 Dkc1 Ppm1j Cmah Rasa3 Fam65b Gzmm Podnl1 Ikbke Il18r1 S1pr5 Dgka Pdlim1 Bin2 Dock11 Cd55 Tcf7 Pde2a Cd84 Traf3ip3 Slc11a2 Kbtbd11 Gramd4 Nsg2 Gab3 Klhl6 Zfp397
