# Supplementary figures and images for: cDC1 Dependent Accumulation of Memory T Cells Is Required for Chronic Autoimmune Inflammation in Murine Testis
Source: Front Immunol. 2021 Jul 26;12:651860. doi: 10.3389/fimmu.2021.651860 (PMC8350123; doi:10.3389/fimmu.2021.651860)

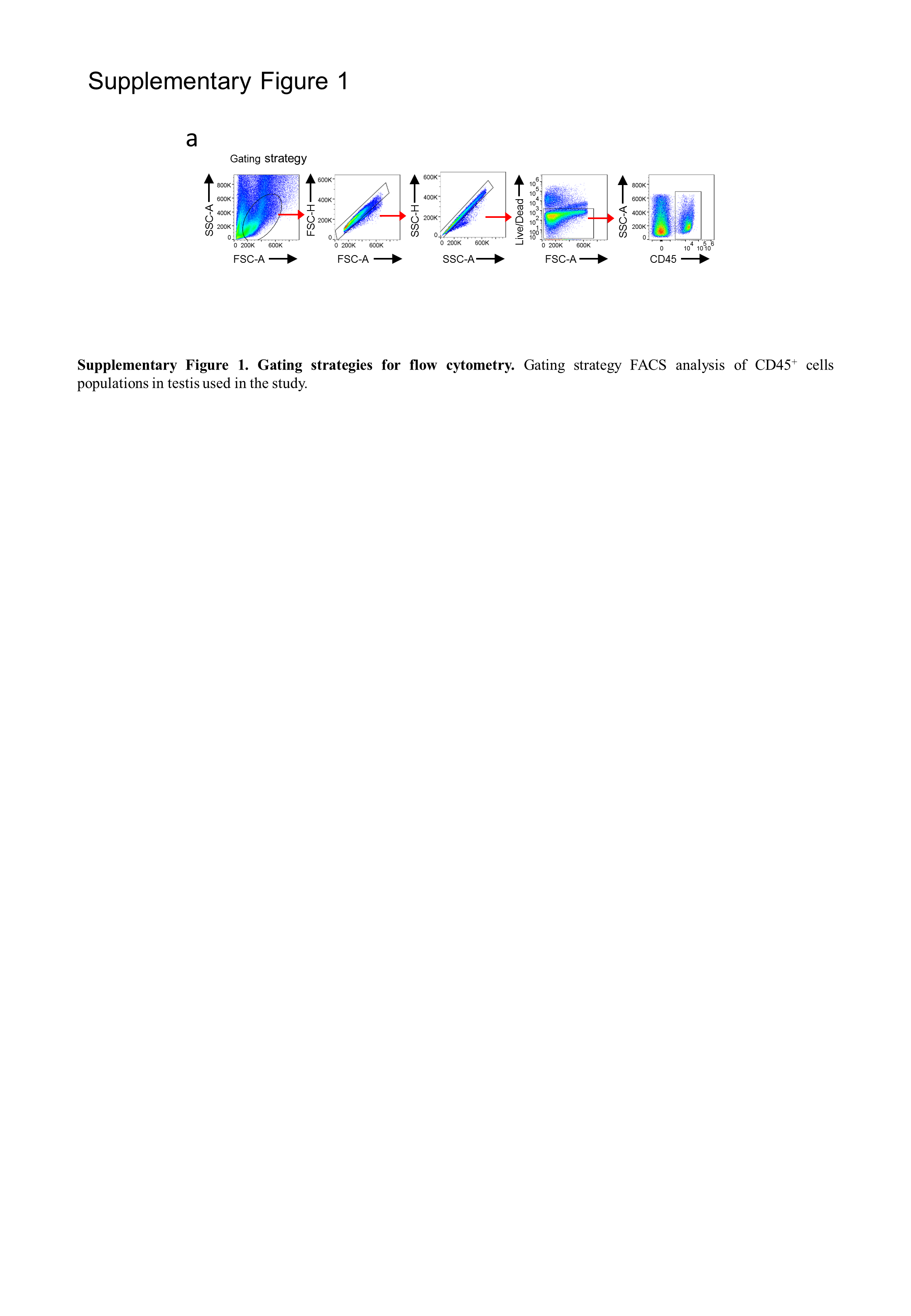

Supplement: Supplementary file 2 [file Image_1.tif]

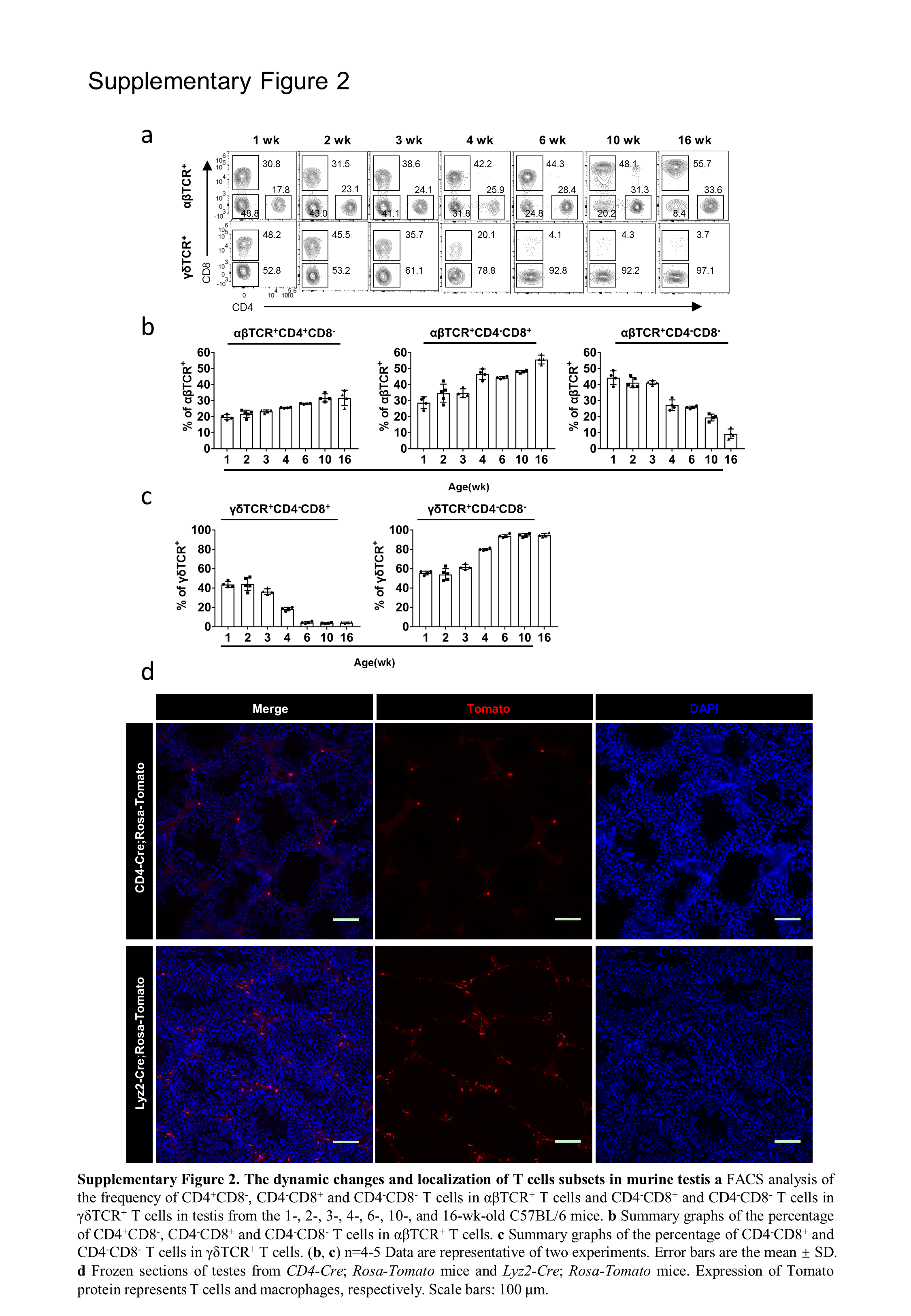

Supplement: Supplementary file 3 [file Image_2.tif]

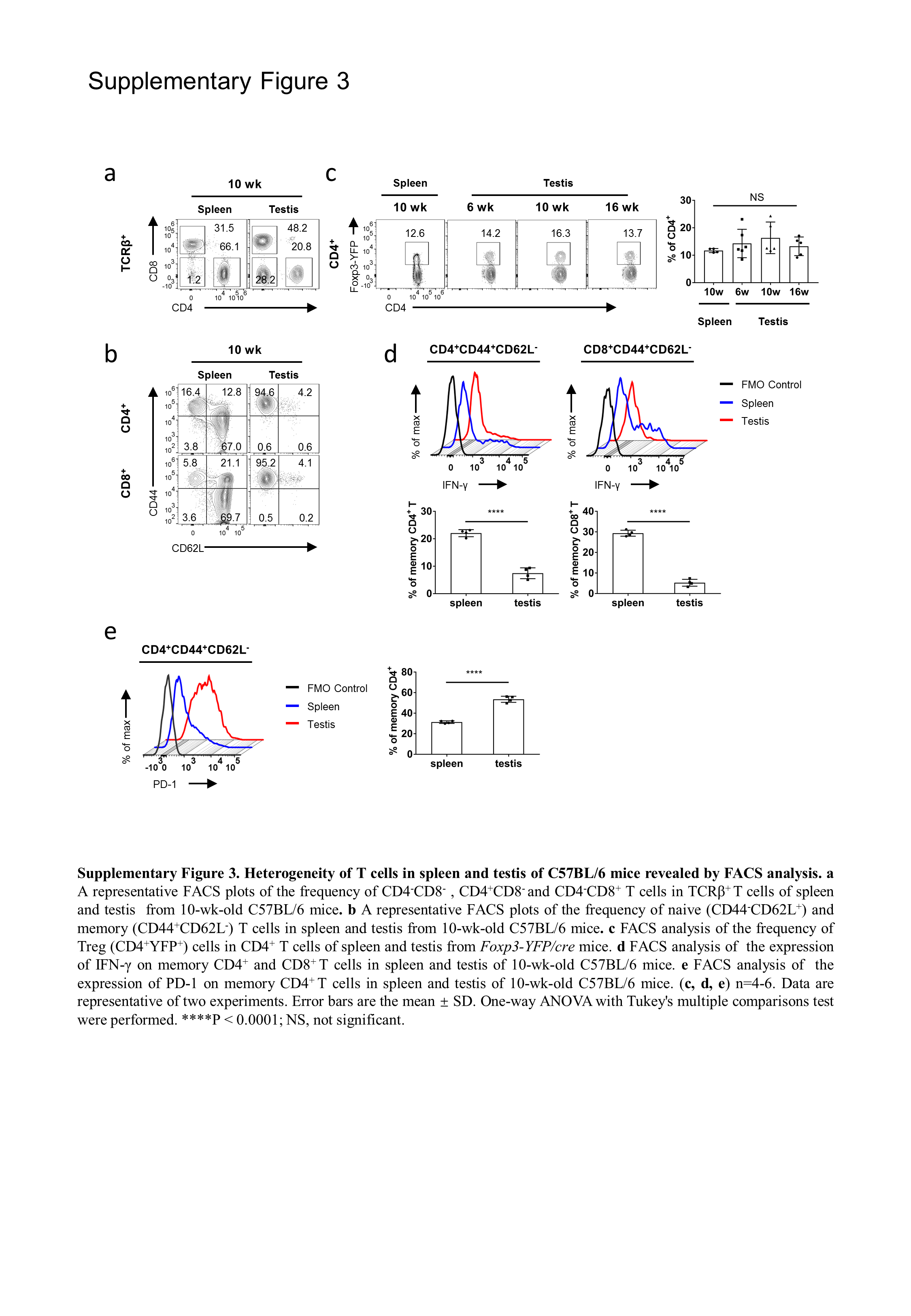

Supplement: Supplementary file 4 [file Image_3.tif]

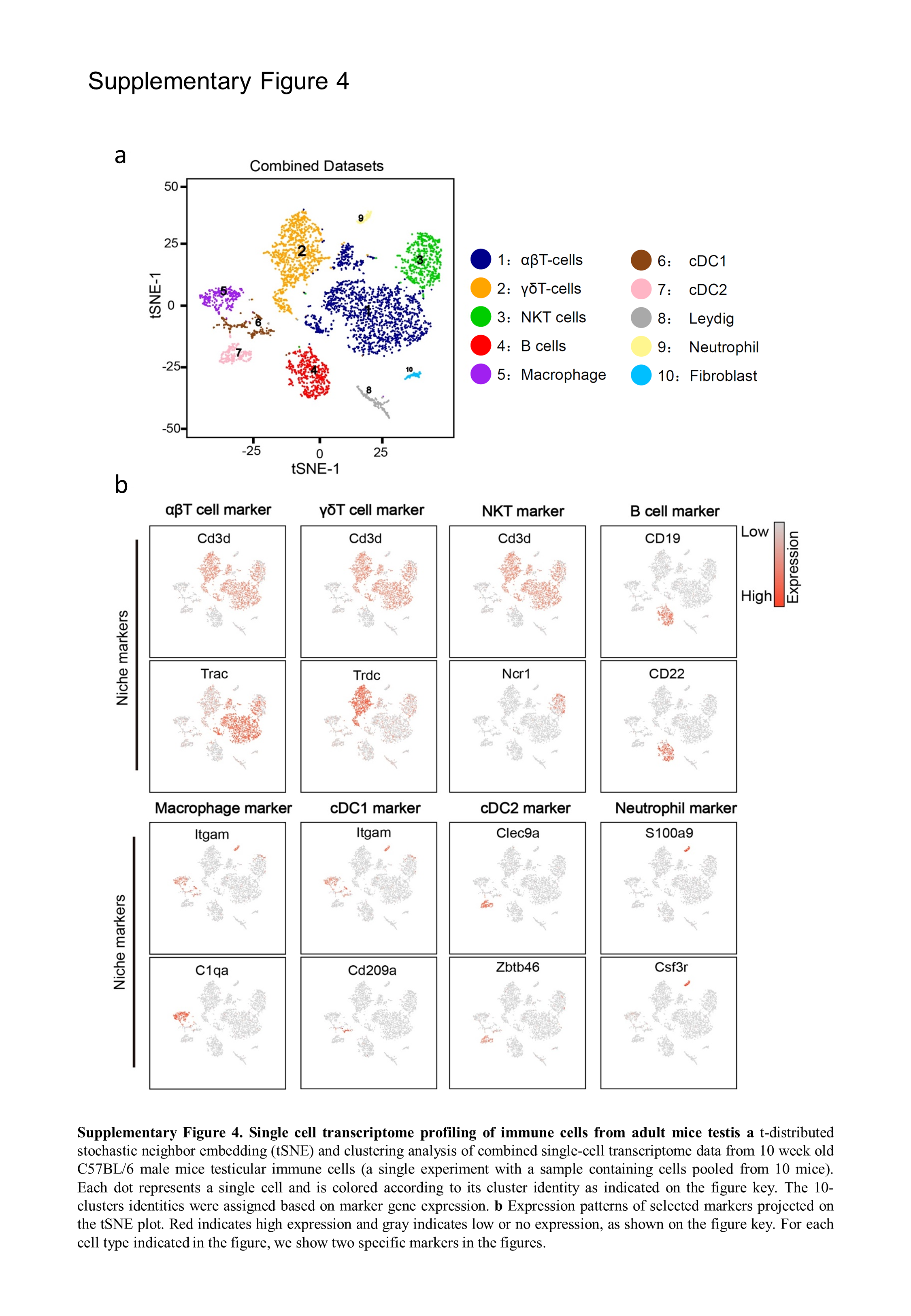

Supplement: Supplementary file 5 [file Image_4.tif]

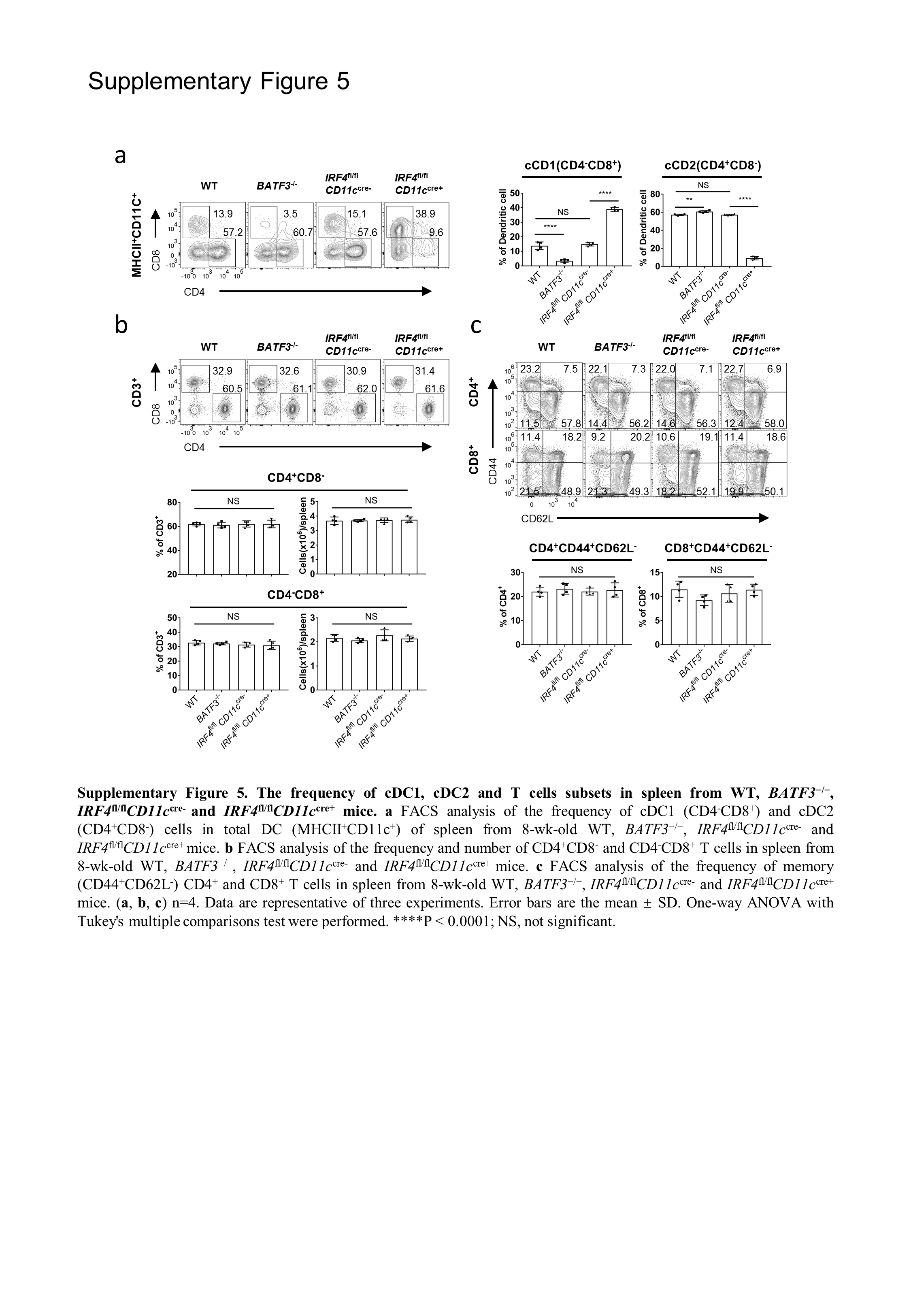

Supplement: Supplementary file 6 [file Image_5.tif]

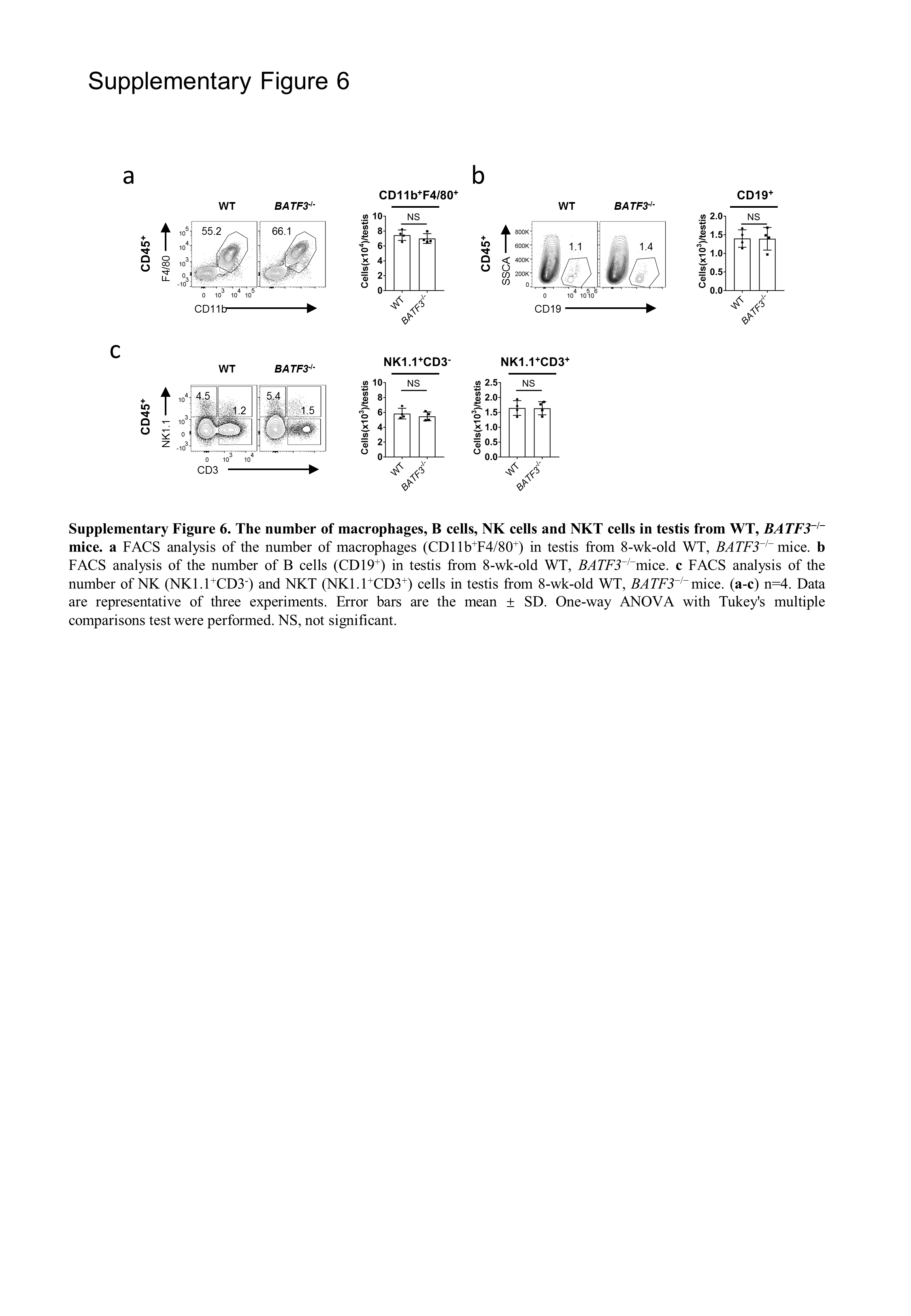

Supplement: Supplementary file 7 [file Image_6.tif]

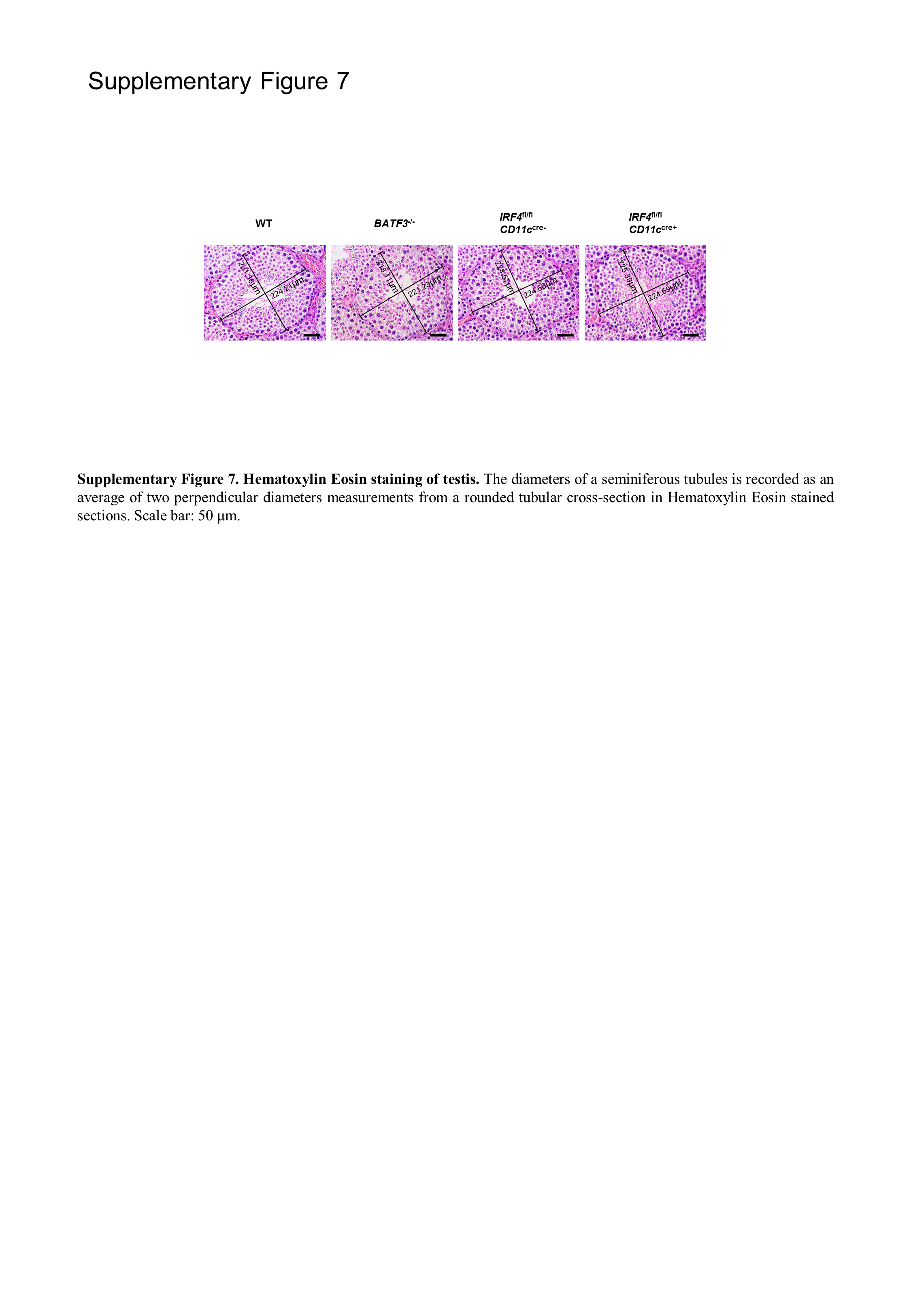

Supplement: Supplementary file 8 [file Image_7.tif]

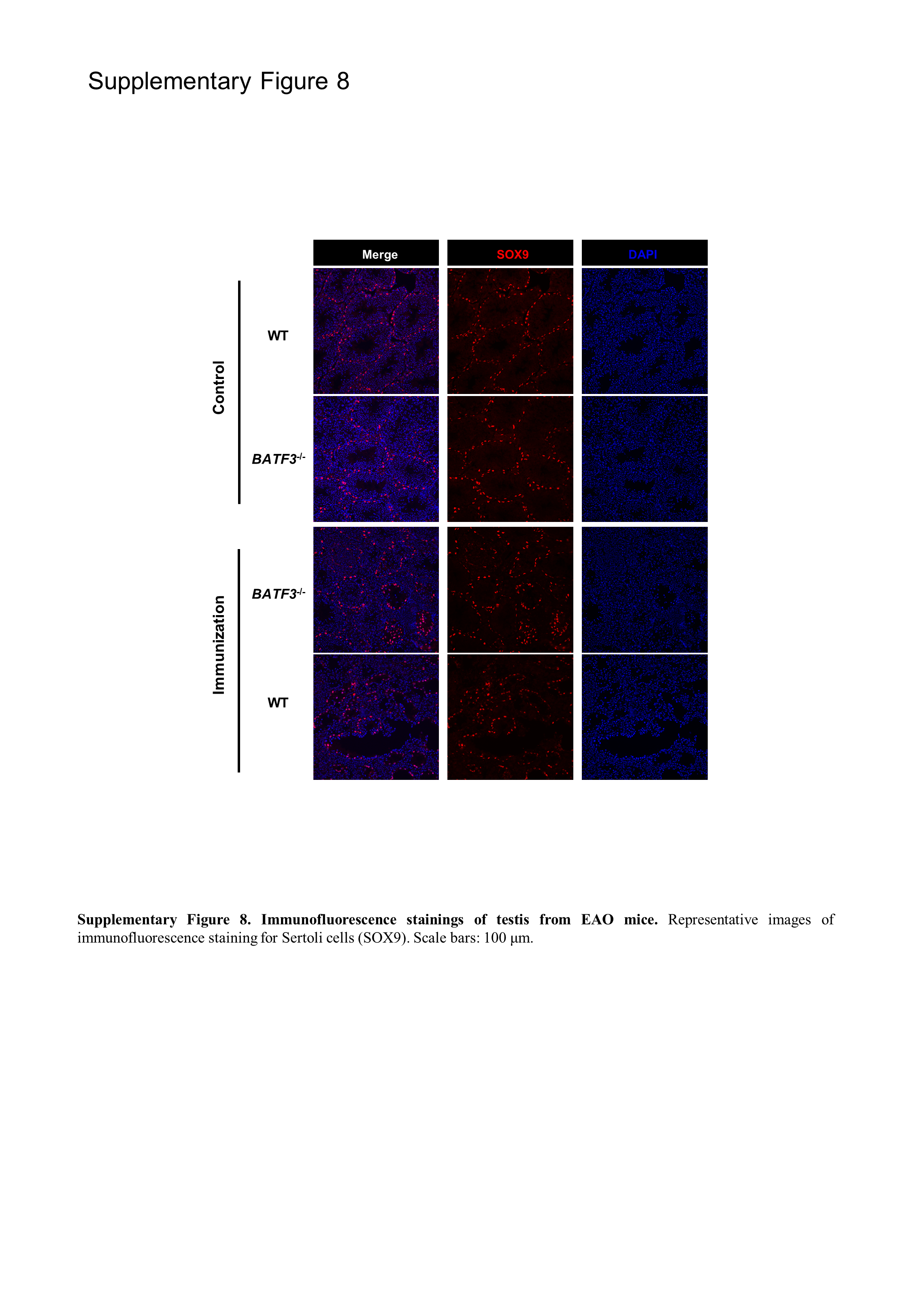

Supplement: Supplementary file 9 [file Image_8.tif]
